# Cleavage of Grb2-Associated Binding Protein 2 by Viral Proteinase 2A during *Coxsackievirus* Infection

**DOI:** 10.3389/fcimb.2017.00085

**Published:** 2017-03-16

**Authors:** Haoyu Deng, Gabriel Fung, Ye Qiu, Chen Wang, Jingchun Zhang, Zheng-Gen Jin, Honglin Luo

**Affiliations:** ^1^Centre for Heart Lung Innovation, St. Paul's Hospital and Department of Pathology and Laboratory Medicine, University of British ColumbiaVancouver, BC, Canada; ^2^Department of Vascular Surgery, RenJi Hospital, Shanghai Jiaotong University School of MedicineShanghai, China; ^3^Institute of Basic Theory for Chinese Medicine, China Academy of Chinese Medical ScienceBeijing, China; ^4^Aab Cardiovascular Research Institute and Department of Medicine, University of Rochester School of Medicine and DentistryRochester, NY, USA

**Keywords:** GAB2, CV-B3, cleavage, virus, viral proteinase 2A

## Abstract

Coxsackievirus type B3 (CV-B3), an enterovirus associated with the pathogenesis of several human diseases, subverts, or employs the host intracellular signaling pathways to support effective viral infection. We have previously demonstrated that Grb2-associated binding protein 1 (GAB1), a signaling adaptor protein that serves as a platform for intracellular signaling assembly and transduction, is cleaved upon CV-B3 infection, resulting in a gain-of-pro-viral-function *via* the modification of GAB1-mediated ERK1/2 pathway. GAB2 is a mammalian homolog of GAB1. In this study, we aim to address whether GAB2 plays a synergistic role with GAB1 in the regulation of CV-B3 replication. Here, we reported that GAB2 is also a target of CV-B3-encoded viral proteinase. We showed that GAB2 is cleaved at G238 during CV-B3 infection by viral proteinase 2A, generating two cleaved fragments of GAB2-N_1−237_ and GAB2-C_238−676_. Moreover, knockdown of GAB2 significantly inhibits the synthesis of viral protein and subsequent viral progeny production, accompanied by reduced levels of phosphorylated p38, suggesting a pro-viral function for GAB2 linked to p38 activation. Finally, we examined whether the cleavage of GAB2 can promote viral replication as observed for GAB1 cleavage. We showed that expression of neither GAB2-N_1−237_ nor GAB2-C_238−676_ results in enhanced viral infectivity, indicating a loss-of-function, rather than a gain-of-function of GAB2 cleavage in mediating virus replication. Taken together, our findings in this study suggest a novel host defense machinery through which CV-B3 infection is limited by the cleavage of a pro-viral protein.

## Introduction

Coxsackievirus serotype B3 (CV-B3) is a non-enveloped, human-pathogenic enterovirus in the *Picornaviridae* family (Luo et al., 2010). It encompasses a 7.4 kb single-stranded, positive-sense RNA genome, and is the most common human pathogen associated with the pathogenesis of pancreatitis, insulin-dependent diabetes mellitus, myocarditis, and aseptic meningitis. Similar to other viruses, CV-B3 evolves diverse mechanisms to manipulate the intracellular signaling pathways to support successful viral infection (Luo et al., 2002; Si et al., 2005; Garmaroudi et al., 2010). We have previously demonstrated that CV-B3 infection induces activation of the mitogen-activated protein kinase (MAPK) signaling pathways (Luo et al., 2002; Si et al., 2005). Inhibition of such activation significantly inhibits CV-B3 infection and reduces virus-mediated pathogenesis (Luo et al., 2002; Si et al., 2005). Recently, we reported that Grb2-associated binding protein 1 (GAB1, NCBI:NP_002030.2), a signaling adaptor protein that acts as a platform for intracellular signaling transduction and assembly, is cleaved upon CV-B3 infection (Deng et al., 2015). As a result, generation of the N-terminal fragment of GAB1 further induces sustained activation of ERK1/2 MAPK and consequent enhancement of viral replication.

GAB2 (NCBI:NP_536739.1) is a functional homolog of GAB1, which also belongs to the family of insulin receptor substrate 1-like multi-substrate proteins and serves as a platform for the assembly of signaling proteins (Holgado-Madruga et al., 1996; Gu et al., 1998; Bouscary et al., 2001; Lock et al., 2002; Chaudhuri et al., 2011). Upon activation by receptor tyrosine kinases, GAB2 undergoes tyrosyl-phosphorylation, creating docking sites for downstream adaptor proteins that mediate further signal transduction. As such, GAB2 has been considered as a major mediator of essential cellular processes, including proliferation, survival, and differentiation. GAB2 is ubiquitously expressed in many organs and depletion of GAB2 has been associated with a severe defect in response to passive allergic challenge and a defective osteoclast differentiation (Nakaoka and Komuro, 2013). The existence of both GAB1 and GAB2 in a single cell raises the question whether each GAB protein mediates specific downstream signaling event upon engagement of different extracellular stimuli, such as growth factors and cytokines (Meng et al., 2005). It is observed that hepatocyte growth factor selectively activates GAB1 in epithelial cells that express both GAB1 and GAB2, partly due to the presence of the Met-binding domain in GAB1 but not in GAB2 (Lock et al., 2002), while Bcr-Abl oncoprotein preferentially utilizes GAB2 as its downstream signaling components in T cells (Gu et al., 1998). Furthermore, signal transduction studies demonstrated that GAB1 and GAB2 have non-redundant roles in vascular endothelial growth factor-mediated migration and survival of endothelial cells (Caron et al., 2009).

In this study, we investigated whether GAB2 plays a synergistic role with GAB1 in controlling CV-B3 replication. We showed that GAB2 was also cleaved upon CV-B3 infection. This cleavage took place at position G238 mediated by virally-encoded proteinase 2A. We further found that knockdown of GAB2 led to reduced production of viral protein and decreased virus titers, suggesting a pro-viral function for GAB2. Moreover, we demonstrated that expression of the cleavage products of GAB2 did not further enhance viral replication, indicating that GAB2 cleavage resulted in its loss-of-function, rather than gain-of-function in supporting viral replication that represents a novel host defense mechanism.

## Methods and materials

### Cell culture and viral infection

HeLa cells purchased from American Type Culture Collection (Manassas, VA, USA) were cultured in Dulbecco's modified Eagle's medium (DMEM) supplemented with 10% fetal bovine serum at 37°C in a humidified incubator with 5% CO_2_. CV-B3 (Nancy strain) was maintained at −80°C freezers. Ultraviolet (UV)-irradiated virus was prepared and viral infection was performed as previously described (Luo et al., 2002).

### Plasmids and siRNAs

Hemagglutinin (HA)-tagged wild-type GAB2 (GAB2^WT^) was a generous gift from Dr. Roger Daly at the Monash University (Melbourne, Australia). The HA-tagged GAB2^G238E^ was established by replacing the glycine (G) residue at amino acid 238 of wild-type GAB2 with glutamic acid (E). The wild-type GAB2 was used as a template to generate two truncated forms of GAB2. The resulting fragments were cloned into a vector expressing 3 × Flag at its N-terminus (3 × Flag-GAB2-N_1−237_ and 3 × Flag-GAB2-C_238−676_). The small interfering RNAs (siRNAs) against human GAB1 and human GAB2 were purchased from Dharmacon (Ottawa, ON, Canada).

### Transient transfection and drug treatment

Transfection of plasmids and siRNAs was performed according to manufacturer's instructions using Lipofectamine® 2000 (#11668019, Invitrogen, Burlington, ON, Canada) for plasmid transfection or Oligofectamine® (#12252-011, Invitrogen, Burlington, ON, Canada) for siRNA transfection. For drug treatment, pan-caspase inhibitor zVAD (50 μM, #550377, BD Biosciences, San Jose, CA, USA) was applied for the blockage of general caspase activation. SB203580 (50 μM, #S8307, Sigma-Aldrich, St. Louis, MO, USA) was used for inhibition of p38 activity.

### Western blot analysis

Cellular proteins were extracted using modified oncogene science lysis buffer (250 mM NaCl, pH 7.2, 50 mM Tris-HCl, 0.1% NP-40, 2 mM EDTA, and 10% glycerol) supplemented with protease inhibitors. The concentrations of the protein were determined by Bradford assay. A total of 20–40 μg of protein per sample was loaded for sodium dodecyl sulfate-polyacrylamide gel electrophoresis. Western blotting was performed as described previously (Shi et al., 2014). Primary antibodies used in this study were: (1) anti-human GAB2 (#3239, Cell signaling, Beverly, MA, USA); (2) anti-Flag (sc-807, Santa Cruz, Dallas, TX, USA); (3) anti-HA (sc-805, Santa Cruz, Dallas, TX, USA); (4) anti-viral capsid protein VP1 (NCL-ENTERO, Leica biosystems, Concord, ON, Canada); (5) anti-cleaved caspase-3 (#9661, Cell signaling, Beverly, MA, USA); (6) anti-phospho-p44/42 MAPK (ERK1/2) (#4094, Cell signaling, Beverly, MA, USA); (7) anti-phospho-p38 MAPK (#4511, Cell signaling, Beverly, MA, USA); (8) anti-phospho-SAPK/JNK (#4668, Cell signaling, Beverly, MA, USA); (9) anti-β-actin (#2228, Sigma-Aldrich, St. Louis, MO, USA); and (10) anti-phospho-HSP27 (sc-81498, Santa Cruz, Dallas, TX, USA).

### *In vitro* cleavage assay

Purified viral proteinase 2A and catalytically inactive 2A were generous gifts from Dr. Eric Jan at the University of British Columbia. The assay was performed according to the protocol previously described (Deng et al., 2015). The cleaved fragments of GAB2 were examined by western blotting. CV-B3-infected HeLa cells were used as a positive control.

### Viral plaque assay

The virus titers in the supernatant of CV-B3-infected cells were measured by plaque assay as described previously (Deng et al., 2015). Briefly, the supernatant was serially diluted and overlaid on a monolayer of HeLa cells with 90% confluence for 1 h prior to the replacement of the medium with complete DMEM containing 0.75% agar. After 3-day incubation, the cells were fixed with Carnoy's fixative (75% ethanol and 25% acetic acid) and then stained with 1% crystal violet. The resulting plaques were counted and the virus titers were calculated as plaque forming unit (PFU) per milliliter.

### Statistical analysis

All results presented are representative of at least three independent experiments. Results are expressed as means ± standard deviations (*SD*). Statistical analysis was performed with unpaired student's *t*-test. The *p* < 0.05 were considered to be statistically significant.

## Results

### Proteolytic process of GAB2 upon CV-B3 infection

**Figure 1:**
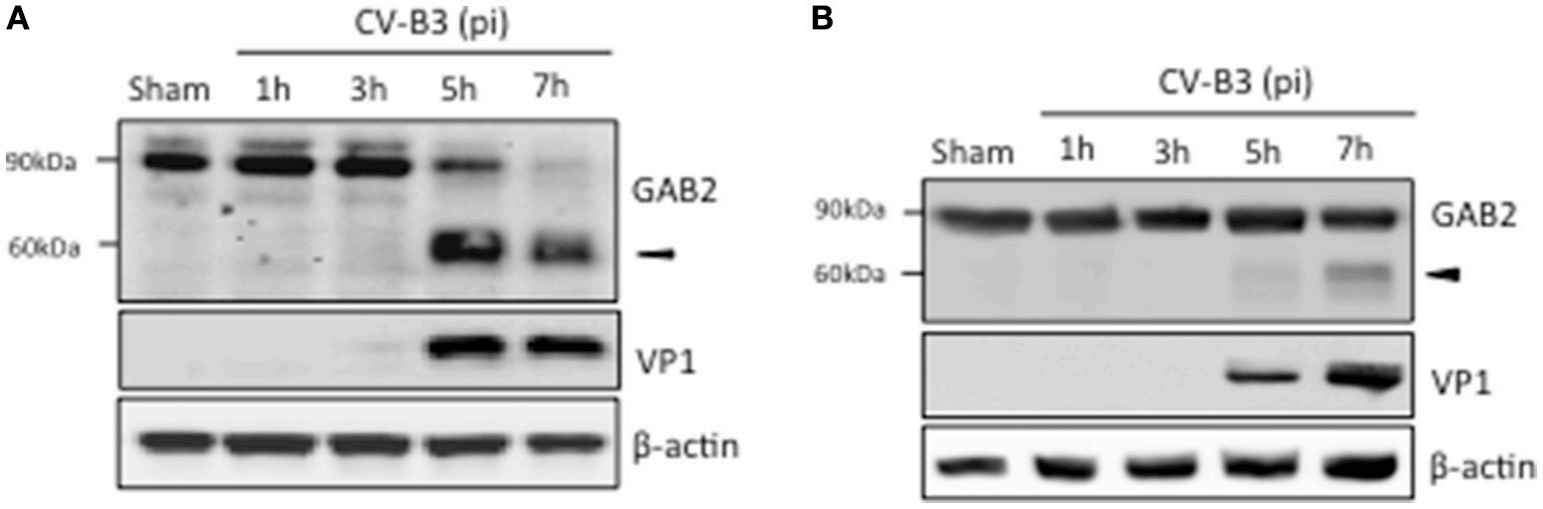
**GAB2 is cleaved upon CV-B3 infection. (A)** HeLa cells were inoculated with CV-B3 at an MOI of 10 for indicated time. Western blotting was conducted to examine protein levels of GAB2 using an antibody that recognizes the C-terminus of GAB2. Viral capsid protein VP1 was probed as an evidence of viral infection and β-actin was examined as protein loading control. **(B)** HeLa cells were transiently transfected with a plasmid expressing wild-type GAB2 with an epitope of HA at its C-terminus for 48 h, followed by CV-B3 infection for various time points as indicated. Western blot analysis was performed for detection of exogenous GAB2 (using anti-HA antibody), VP1, and β-actin.

To understand the possible role of GAB2 in CV-B3 infection, we first examined the protein level of GAB2 upon viral infection. As shown in Figure 1A, protein level of GAB2 began to decrease at 5 h and disappeared at 7 h post-infection, accompanied by the generation of an additional band at around 60 kDa using an antibody against the C-terminus of GAB2, suggesting a possible cleavage event. To verify this, a plasmid expressing C-terminal HA-tagged GAB2 (GAB2-HA) was utilized. HeLa cells transfected with GAB2-HA were infected with CV-B3 for indicated hours and protein expression of GAB2 was examined. We found that, similar to the finding in Figure 1A, an extra band of exogenous GAB2 was detected at around 60 kDa using an anti-HA antibody (Figure 1B), indicating that GAB2 is also cleaved after CV-B3 infection. It is noted that the cleavage efficiency of exogenously transfected GAB2 is much lower than that of endogenous GAB2. We have previously reported that liposome-mediated cDNA transfection inhibits CV-B3 attachment to the cells by disrupting membrane cholesterol (Wong et al., 2006). Thus, the decreased cleavage efficiency is likely a result of reduced viral infectivity in transfected cells.

### CV-B3 proteinase 2A-mediated cleavage of GAB2

**Figure 2:**
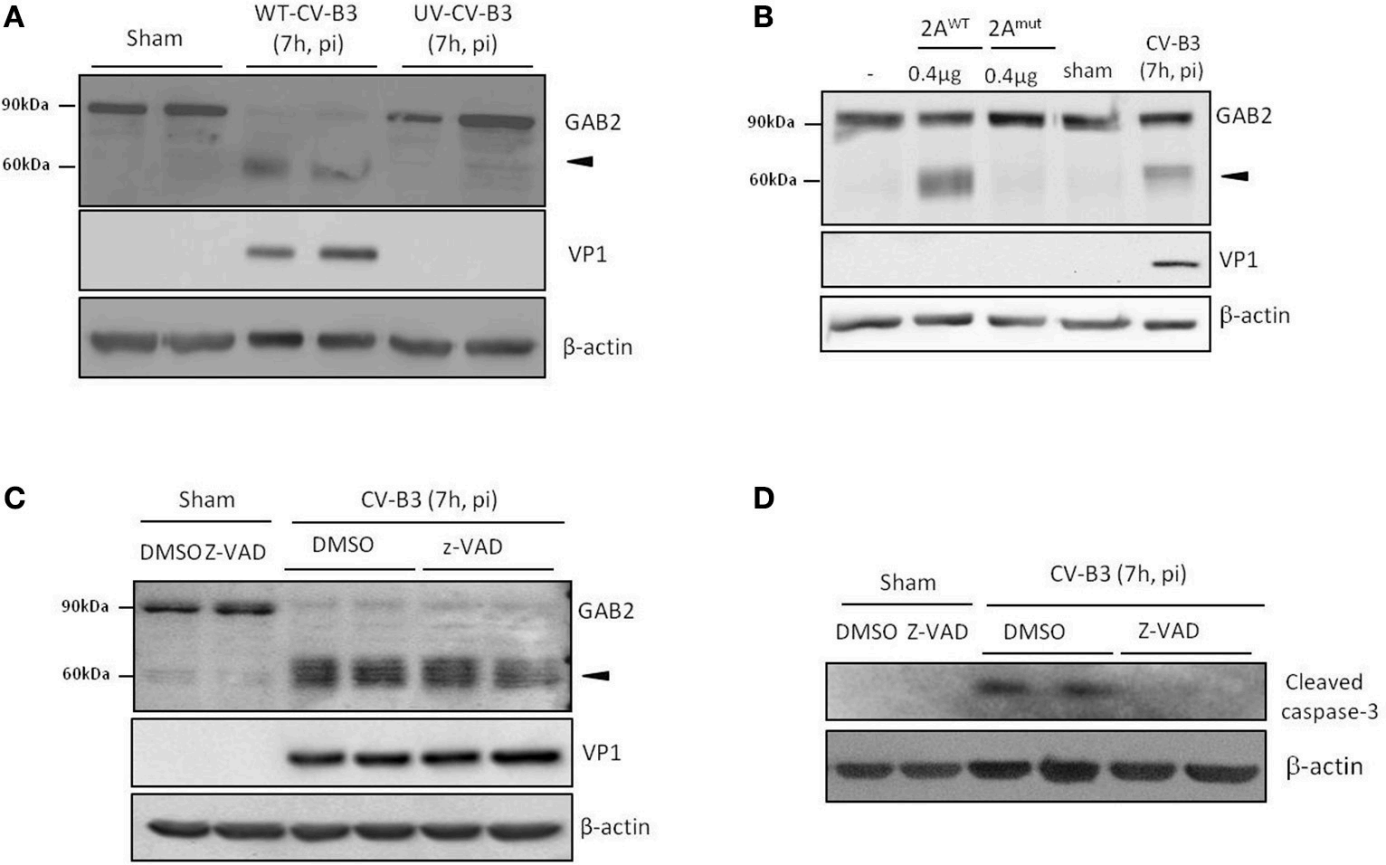
**Cleavage of GAB2 is catalyzed by CV-B3-encoded proteinase 2A. (A)** HeLa cells were sham-infected or infected with either wild-type (WT) or ultraviolet (UV)-irradiated CV-B3 at an MOI of 10 for 7 h. Protein levels of GAB2, VP1, and β-actin were detected by western blot analysis. **(B)** HeLa cells were transiently transfected with GAB2-hemagglutinin (HA) for 48 h. Fifty micrograms of cell lysates were incubated with 0.4 μg of purified CV-B3 wild-type (2A^WT^) or catalytic mutant (2A^Mut^) of 2A for 18 h. Western blotting was carried out to examine protein expression of GAB2 using an anti-HA antibody. Sham and CV-B3-infected, GAB2-HA-transfected HeLa cell lysates were loaded as negative and positive control (right two lanes), respectively. **(C,D)** HeLa cells were sham or CV-B3-infected in the presence of pan-caspase inhibitor, z-VAD (50 μM), or vehicle (DMSO) for 7 h. Western blotting was performed and protein levels of GAB2, VP1, and β-actin were examined **(C)**. The inhibition of caspase activation by z-VAD was confirmed by the blockage of caspase-3 cleavage **(D)**.

We then investigated the mechanism leading to GAB2 cleavage. We first tested whether viral replication is required for GAB2 cleavage. We utilized UV-irradiated viruses, which are capable of interacting with viral receptor and subsequent entering into cells, but unable to replicate. We showed that GAB2 was not cleaved in cells infected with UV-CV-B3, suggesting that GAB2 cleavage is dependent on viral replication (Figure 2A).

CV-B3 encodes two proteinases, 2A and 3C, which not only process viral polyprotein into individual structural and nonstructural protein, but also target cellular proteins to either facilitate infection or provoke host anti-viral machinery (Jurgens et al., 2006; Zaragoza et al., 2006; Feng et al., 2014). *In vitro* cleavage assay was performed to determine whether viral proteinases contribute to the cleavage of GAB2 upon CV-B3 infection. As shown in Figure 2B, incubation with wild-type 2A (2A^WT^) led to the generation of a cleavage band at ~60 kDa, corresponding to what was detected during CV-B3 infection. However, the catalytic mutant of 2A (2A^mut^) failed to cleave GAB2, suggesting that cleavage of GAB2 is mediated through the catalytic activation of 2A. *In vitro* cleavage assay was also conducted using purified 3C proteinase. However, no cleavage products were detected (data not shown).

Furthermore, to rule out the possible role of caspase activation in GAB2 cleavage, we treated cells with the general caspase inhibitor, z-VAD. We found that caspase inhibition did not prevent the cleavage of GAB2 (Figure 2C). The inhibition of caspase activity by z-VAD was confirmed by the blockage of caspase-3 cleavage (Figure 2D). Taken together, our results suggested that the cleavage of GAB2 detected during CV-B3 infection is mediated *via* the catalytic activity of viral proteinase 2A, independent of the activation of caspase.

### Cleavage of GAB2 between H237 and G238

**Figure 3:**
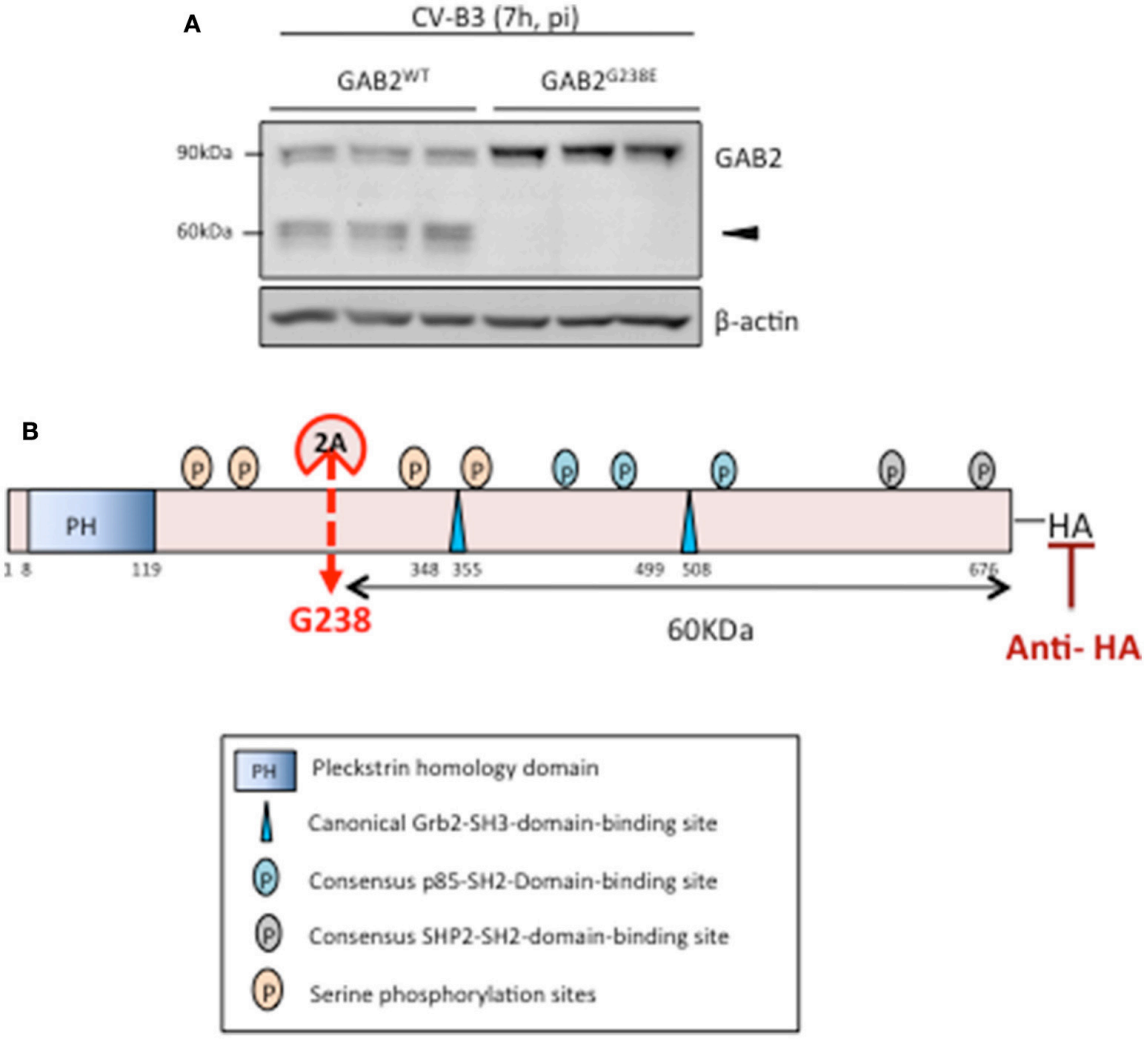
**GAB2 is cleaved between H237 and G238 during CV-B3 infection. (A)** HeLa cells were transiently transfected with either GAB2^WT^-HA or GAB2^G238E^-HA for 48 h, followed by CV-B3 infection for 7 h. Cleavage of GAB2 was examined using anti-HA antibody by western blot analysis. Protein levels of β-actin were examined as loading control. **(B)** Schematic diagram of different function domains and cleavage site of GAB2.

Based on the reported cleavage consensus motif of proteinase 2A (i.e., L/I/MxT/S/Nx//G, // indicates the scissile bond, P4 position—L (leucine), I (isoleucine), or M (methionine), P2 position—T (threonine), S (serine), or N (asparagine), P1' position is commonly G (glycine), x indicates any amino acid residues) (Wong et al., 2012), we found one potential cleavage sequence (^234^LASHG^238^) on GAB2. To determine whether GAB2 is cleaved at this site, we performed site-directed mutagenesis to replace G at position 238 with glutamic acid (E). As shown in Figure 3A, GAB2^G238E^ mutant was uncleavable upon CV-B3 infection, suggesting that G238 is the cleavage site. Figure 3B illustrates the functional domains, the cleavage site, and the resulting cleavage fragments of GAB2 following CV-B3 infection.

### Inhibition of viral replication and MAPK signaling by knocking down GAB2

**Figure 4:**
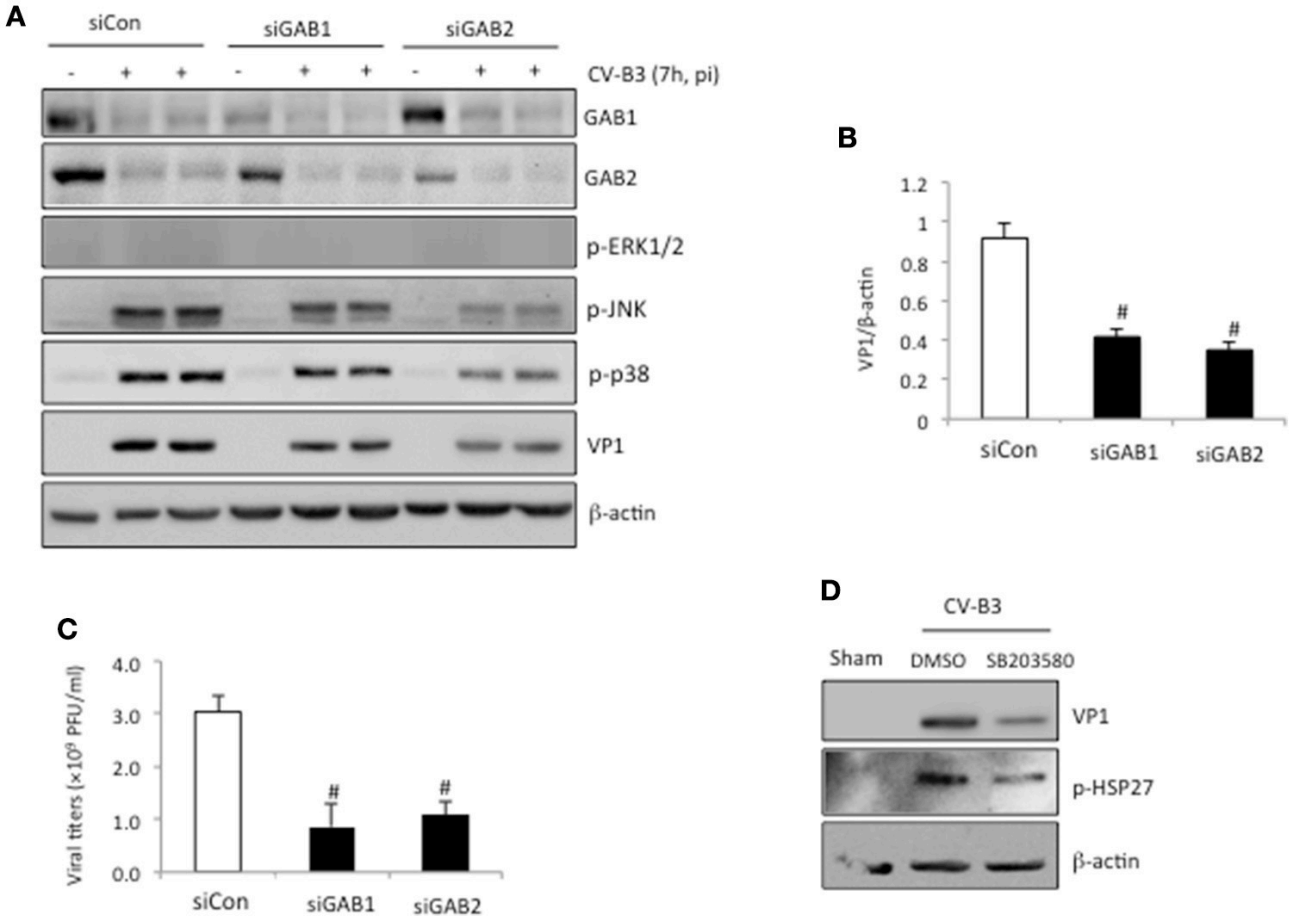
**Knockdown of GAB2 inhibits viral replication and MAPK signaling. (A)** HeLa cells were transiently transfected with control siRNA (siCon), GAB1- or GAB2-targeting siRNA (siGAB1 or siGAB2) as indicated for 48 h, followed by CV-B3 infection at an MOI of 10 for 7 h. Western blotting was performed to examine protein levels of GAB1, GAB2, VP1, p-ERK1/2, p-p38, p-JNK, and β-actin. **(B)** Protein levels of VP1 were quantitated by densitometric analysis, normalized to β-actin (mean ± *SD, n* = 3). ^#^*p* < 0.001 compared to siCon. **(C)** Virus titers in the supernatant collected from the experiments above were measured by plaque assay. The virus titers are presented as mean ± *SD* (*n* = 4). ^#^*p* < 0.001 compared to siCon. **(D)** HeLa cells were sham or CV-B3 infected in the presence of p38 inhibitor (SB203580, 50 μM) or vehicle (DMSO) for 7 h. Western blotting was performed to examine protein levels of p-HSP27, VP1, and β-actin.

We have previously shown that knockdown of GAB1 inhibits CV-B3 replication (Deng et al., 2015). Here, we questioned whether GAB2 plays a similar role in CV-B3 infection. Figure 4 revealed that gene-silencing of GAB2 resulted in significant decreases in viral protein production (Figures 4A,B) and virus titers (Figure 4C), accompanied by a large reduction of CV-B3-induced phosphorylation of JNK and p38 (Figure 4A), suggesting a pro-viral function for GAB2. The importance of p38 activation in viral replication was further demonstrated using a selective p38 inhibitor (SB203580). Figure 4D showed that treatment with SB203580 led to a marked reduction of viral protein expression. Inhibition of p38 activity by SB203580 was confirmed by reduced levels of phospho-HSP27, a downstream target of p38 (Figure 4D). No significant effects of JNK inhibitor on viral replication were observed (data not shown). Interestingly, unlike deletion of GAB1 that caused a decrease in phosphorylated ERK1/2, knockdown of GAB2 had no effect on virus-mediated ERK1/2 phosphorylation (Figure 4A). We further examined the impacts of forced expression of exogenous GAB2 on viral replication. We found that viral protein levels and viral loads failed to further increase in cells overexpressing GAB2 (data not shown), indicating that the levels of endogenous GAB2 are already saturated for viral replication.

### Cleavage of GAB2 does not further increase viral replication

**Figure 5:**
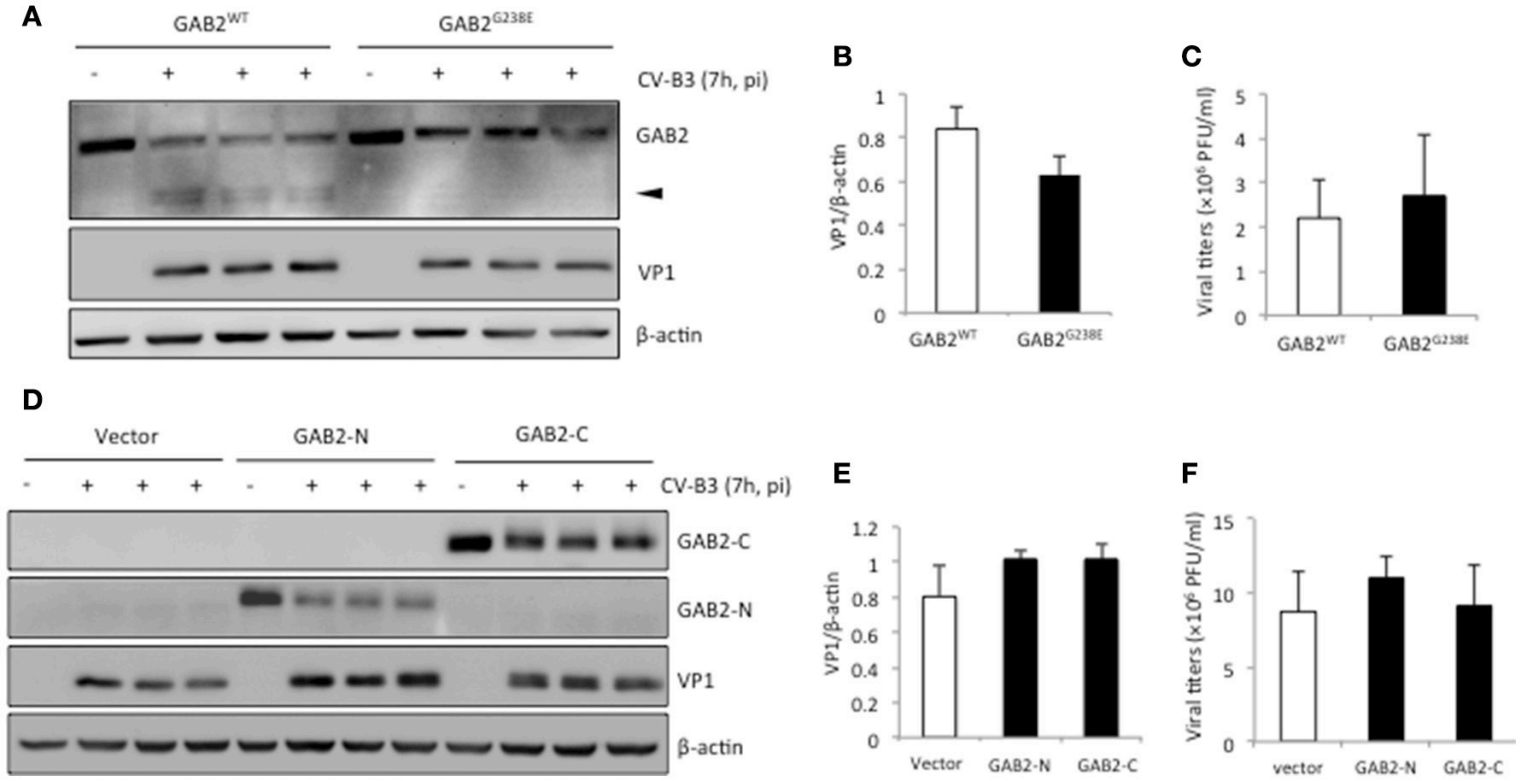
**Cleavage of GAB2 does not further increase viral replication**. HeLa cells were transiently transfected with **(A)** C-terminal HA-tagged GAB2^WT^ or non-cleavable GAB2 mutant (GAB2^G238E^), or **(D)** empty vector, N-terminal Flag-tagged GAB2-N, or GAB2-C for 24 h, followed by CV-B3 infection for 7 h. Cell lysates were collected for western blot analysis of protein levels of GAB2^WT^–HA or GAB2^G238E^-HA using anti-HA antibody, Flag-GAB2-N, or Flag-GAB2-C using anti-Flag antibody. **(B,E)** Protein levels of VP1 in **(A)** and **(D)** were quantitated by densitometric analysis, normalized to β-actin (mean ± *SD, n* = 3). **(C,F)** The supernatant from above experiments was harvested for plaque assay, and the virus titers are presented as mean ± *SD* (*n* = 3).

We next sought to determine the consequence of GAB2 cleavage during viral infection. HeLa cells were either transfected with GAB2^WT^ or non-cleavable GAB2 (GAB2^*G*238*E*^). We showed that the levels of viral protein (Figures 5A,B) and virus titers (Figure 5C) were comparable between cells expressing GAB2^WT^ and GAB2^G238E^, suggesting that cleavage of GAB2 had no direct benefits to virus replication. Moreover, we examined the influence of overexpression of either GAB2-N or GAB-C on viral replication. As shown in Figures 5D–F, neither VP1 levels nor virus titers were significantly altered in cells expressing GAB2 cleavage fragments compared with empty vector control. Collectively, our results suggest that cleavage of GAB2 results in the loss of its function in promoting viral infection, rather than the gain of a pro-viral activity.

## Discussion

GAB2 is a scaffolding/docking protein that transduces cellular signals from the receptors to the intracellular downstream molecules. Upon activation by a variety of extracellular stimuli, such as growth factors and cytokines, GAB2 translocates from the cytoplasm to the plasma membrane, where it is tyrosine-phosphorylated by receptor tyrosine kinases and then recruits several Src-homology-2 (SH2)-containing proteins, including SH2-containing protein tyrosine phosphatase-2 (SHP2), Phosphatidylinositol 3-kinase (PI3K), and Crk, subsequently leading to the activation of multiple downstream signaling pathways (e.g., MAPKs and Akt) that are critical for cell proliferation, differentiation, apoptosis, and survival (Holgado-Madruga et al., 1996; Gu et al., 1998; Chaudhuri et al., 2011).

It has been well-documented that activation of the ERK1/2 MAPK promotes CV-B3 replication (Luo et al., 2002; Opavsky et al., 2002; Cunningham et al., 2003; Jensen et al., 2013). Inhibition of this pathway by either chemical inhibitors or a dominant-negative construct significantly decreases the production of viral protein and progeny virion (Luo et al., 2002; Cunningham et al., 2003; Jensen et al., 2013; Opavsky et al., 2002). We have previously revealed an important role for GAB1-mediated ERK1/2 activation in CVB3 infection (Deng et al., 2015). However, in contrast to gene-silencing of GAB1, in this study we found that knockdown of GAB2 had no effect on ERK1/2 phosphorylation, suggesting a pro-viral activity for GAB2 independent of the ERK1/2 pathway. In addition to the ERK1/2 pathway, it has been previously shown that the p38 MAPK also plays a critical role in CV-B3 infection through facilitating viral spread and propagation (Si et al., 2005; Marchant et al., 2009; Jensen et al., 2013). The significance of p38 pathway in viral infection is confirmed in the current study (Figure 4D). Thus, the pro-viral function of GAB2 is likely executed by activating the p38 pathway, rather than the ERK1/2 pathway. It is also postulated that cleavage of GAB2 contributes to an antiviral mechanism via inhibition of the p38 MAPK activity. In addition, we speculate that the inability of GAB2 knockdown to block ERK1/2 phosphorylation is due to a compensatory function of GAB1 in ERK1/2 activation.

We have previously shown that the N-terminal cleavage fragment of GAB1 (GAB1-N_1−174_) further enhances ERK1/2 activation and facilitates viral growth (Deng et al., 2015). However, this effect was not detected for GAB2-N_1−237_. Although GAB1-N_1−174_ and GAB2-N_1−237_share a highly conserved PH domain (~67% identity), GAB2-N_1−237_ appears to contain distinct and additional docking sites, which likely result in a differential preference for harboring downstream signaling molecules.

Enteroviruses encode two proteinases, 2A and 3C, whose primary function is to process the viral polyprotein. In addition, they also target specific host proteins central for protein translation, RNA replication/stability, signal transduction, and the maintenance of normal cellular structure, to facilitate viral infection (Ventoso et al., 1998; Barco et al., 2000; Li et al., 2001; Jurgens et al., 2006; Kempf and Barton, 2008). For example, CV-B3-encoded proteinases have been shown to cleave host eukaryotic initiation factor 4G (eIF4G) and poly (A)-binding protein (PABP) to shutoff cap-dependent host protein synthesis, while allowing cap-independent, IRES (internal ribosome entry site)-mediated viral mRNA translation (Lamphear et al., 1993; Kerekatte et al., 1999). It has also been reported that CV-B3 proteinases disrupt innate immune response *via* cleaving melanoma differentiation-associated protein 5 and mitochondrial antiviral signaling protein, permitting viral escape from the host immune surveillance (Mukherjee et al., 2011; Feng et al., 2014). In this study, we demonstrate that GAB2 is cleaved following CV-B3 infection by viral proteinase 2A and knockdown of GAB2 results in reduced viral replication, supporting an pro-viral activity for GAB2. Why does CV-B3 induce the cleavage of a protein that promotes its infection? We postulate that GAB2 is an innocent bystander of proteinase 2A. It is conceivable that any proteins comprising a consensus cleavage recognition motif, such as GAB2, could possibly be targeted by viral proteinases. Indeed, it has been previously shown that CV-B3 infection results in the cleavage of inhibitor of κBα (IκBα) to generate a proteolytic fragment that subsequently limits viral replication, and thus cleavage of IκBα is regarded as a crucial step for the host to recognize and respond to the pathogens (Zaragoza et al., 2006).

To establish a causal relationship between GAB2 cleavage and viral infection, one could test whether expression of a non-cleavable GAB2 can enhance viral replication. However, in this study, we showed that overexpression of a non-cleavable form of GAB2 (GAB2^G238E^) failed to further increase viral replication compared to GAB2^WT^-transfected cells (Figures 5A–C). This is likely due to the fact that HeLa cells express high-levels of endogenous GAB2, obscuring the role of exogenous GAB2 in viral replication. In addition, as mentioned earlier, a large portion of the exogenously transfected GAB2^WT^ remains uncleaved following infection, further attenuating the difference in viral replication between GAB2^WT^- and GAB2^G238E^-transfected cells.

In Figure 5D, we showed that the protein levels of GAB2-N and GAB2-C were markedly reduced following viral infection. There are two possible explanations for this observation. First, CV-B3 infection results in the shutoff of cap-dependent host protein translation as a result of viral proteinase-mediated cleavage of eIF4G and PABP as discussed above (Lamphear et al., 1993; Kerekatte et al., 1999). Thus, upon CV-B3 infection, a decrease in protein synthesis of transfected GAB2-N and GAB2-C is expected. Second, at the late stage of viral infection, cellular proteinases are activated (Carthy et al., 1998), which further contributes to the reduced expression of GAB2 fragments.

In conclusion, our findings in this study that GAB2 is cleaved upon CV-B3 infection through the proteolytic activity of virus-encoded proteinase 2A represent a novel host anti-viral strategy against CV-B3 infection.

## Author contributions

Design the experiments: HD, HL, ZJ. Conduct the experiments: HD, GF, YQ, CW, JZ. Write the manuscript: HD, HL.

## Funding

This work was supported by the Canadian Institutes of Health Research (MOP-119274 to HL) and the Heart and Stroke Foundation of Canada (G-16-00013800 to HL). HD is the recipient of a four-year Ph.D. fellowship from the University of British Columbia.

### Conflict of interest statement

The authors declare that the research was conducted in the absence of any commercial or financial relationships that could be construed as a potential conflict of interest. The reviewer RPK and handling Editor declared their shared affiliation and the handling Editor states that the process nevertheless met the standards of a fair and objective review.
